# Dendritic inhibition mediated by O-LM and bistratified interneurons in the hippocampus

**DOI:** 10.3389/fnsyn.2014.00023

**Published:** 2014-09-30

**Authors:** Christina Müller, Stefan Remy

**Affiliations:** ^1^Neuronal Networks Group, German Center for Neurodegenerative Diseases within the Helmholtz AssociationBonn, Germany; ^2^Department of Epileptology, University of BonnBonn, Germany

**Keywords:** oriens-lacunosum moleculare interneuron, bistratified interneuron, dendritic inhibition, theta oscillation, modulation

## Abstract

In the CA1 region of the hippocampus pyramidal neurons and GABAergic interneurons form local microcircuits. CA1 interneurons are a diverse group consisting of many subtypes, some of which provide compartment-specific inhibition specifically onto pyramidal neuron dendrites. In fact, the majority of inhibitory synapses on pyramidal neurons is found on their dendrites. The specific role of a dendrite-innervating interneuron subtype is primarily determined by its innervation pattern on the distinct dendritic domains of pyramidal neurons. The efficacy of dendritic inhibition in reducing dendritic excitation depends on the relative timing and location of the activated excitatory and inhibitory synapses. *In vivo,* synaptic properties such as short-term plasticity and neuro-modulation by the basal forebrain, govern the degree of inhibition in distinct dendritic domains in a dynamic, behavior dependent manner, specifically during network oscillation such as the theta rhythm. In this review we focus on two subtypes of dendrite-innervating interneurons: the oriens-lacunosum moleculare (O-LM) interneuron and the bistratified interneuron. Their molecular marker profile, morphology, and function *in vivo* and *in vitro* are well studied. We strive to integrate this diverse information from the cellular to the network level, and to provide insight into how the different characteristics of O-LM and bistratified interneurons affect dendritic excitability, network activity, and behavior.

## INTRODUCTION

Interneurons in the hippocampus play a fundamental role in rhythmic oscillations, of which the theta rhythm (4–10 Hz) is the most prominent (Jung and Kornmüller, 1938; Green and Arduini, 1954; Buzsáki, 2002; Klausberger and Somogyi, 2008; Forro et al., 2013). Remote input from the medial septal nucleus and the diagonal band of Broca (MSDB) to hippocampal interneurons drives theta oscillations, which are predominantly observed during movement, rapid eye movement (REM) sleep and states of arousal (Petsche et al., 1962; Jouvet, 1969; Vanderwolf, 1969; O’Keefe, 1976). In early recordings of hippocampal electroencephalography (EEG) and single units, local GABAergic interneurons were described as “theta cells” due to the phase relationship of their firing to theta oscillation (Ranck, 1973). GABAergic interneurons in the CA1 region of the hippocampus are a highly diverse group targeting CA1 pyramidal neurons and/or other interneurons (Freund and Buzsáki, 1996; Somogyi and Klausberger, 2005). A main parameter used for classification of CA1 interneurons that target pyramidal neurons is the specific subcellular domain on which they form GABAergic synapses.

For example, basket cells extend their axonal arbor to the stratum pyramidale (s.p.). In fact, pyramidal neuron somata are contacted extensively by GABAergic synapses from these cells forming characteristic basket-like structures (Sik et al., 1995; Megías et al., 2001; Somogyi and Klausberger, 2005). This strategic perisomatic location of basket cell synapses, where the input to the cell has to pass after dendritic processing, allows them to exert influence on all inputs received by pyramidal dendrites. Thus basket cells are thought to control the ability of inputs to generate action potential output and are able to synchronize the firing of the pyramidal neuron population (Cobb et al., 1995; Miles et al., 1996). Chandelier or axo-axonic cells are another example of an interneuron subtype characterized by its highly specific innervation pattern. As the names suggest, their terminals on the axon initial segments of pyramidal neurons look like rows of candles on a chandelier (Szentágothai and Arbib, 1974; Szentágothai, 1975; Howard et al., 2005). By innervating ∼2500 pyramidal neurons they also contribute to the output synchronization of CA1 principal cells (Li et al., 1992).

In contrast to the nearly exclusive GABAergic innervation of the somata and axon initial segments, inhibitory and excitatory synapses intermingle on pyramidal neuron dendrites. Despite this lower relative density of GABAergic synapses, 92% of all inhibitory synapses are found on pyramidal neuron dendrites (Megías et al., 2001). In the CA1 region, at least 12 interneuron subtypes, roughly sorted into four groups, can be classified as dendrite-targeting (Klausberger, 2009). The first group consists of oriens-lacunosum moleculare (O-LM) and bistratified interneurons, which both express parvalbumin (PV) and somatostatin (SST). Interneurons of the second group express cholecystokenin (CCK) including three interneuron subtypes, e.g., Schaffer collateral associated cells. Neurogliaform and ivy-cells form the third group and are characterized by a very dense axonal plexus. The fourth group consists of five different long-range GABAergic projection neurons. The specific function of all these dendrite-innervating interneurons in the hippocampal network is yet not fully understood. In this review, we focus on O-LM and bistratified cells comprising the first group of dendrite-targeting interneurons (Klausberger, 2009). O-LM and bistratified cells fire in phase with theta oscillations and are most likely targeted by afferents from the medial septal region. This region plays a crucial role for hippocampal rhythm generation in the behaving animal (Freund and Antal, 1988; Gulyás et al., 1990; Buzsáki, 2002; Klausberger, 2009; Leão et al., 2012; Lovett-Barron et al., 2014). Thus, O-LM and bistratified cells are likely to mediate network-state dependent inhibition on specific parts of pyramidal neuron dendrites.

Excitatory inputs from the two main pathways, the perforant path and the Schaffer collaterals, terminate on distinct CA1 pyramidal neuron dendritic domains. The perforant path terminates on the distal tuft dendrites of CA1 pyramidal neurons, while Schaffer collaterals target the more proximal radial oblique and basal dendrites in stratum radiatum (s.r.) and stratum oriens (s.o.; Amaral and Witter, 1989). O-LM and bistratified cells counteract the excitation in these domains by providing GABAergic inhibition onto the distal dendritic tuft or the proximal dendrites of the pyramidal neurons, respectively (Klausberger, 2009).

Excitatory inputs onto CA1 pyramidal neuron dendrites integrate linearly or supralinearly, depending on active dendritic properties and the clustering of inputs in time and space (Stuart et al., 1997; Golding and Spruston, 1998; Gasparini et al., 2004; Losonczy and Magee, 2006). The activation of inhibitory inputs reduces neuronal excitability by hyperpolarizing the membrane potential and increasing membrane conductance (Koch et al., 1983; Glickfeld et al., 2009). This affects linear integration of excitatory inputs by reducing the gain and/or changing the offset of the input–output function (Mitchell and Silver, 2003; Isaacson and Scanziani, 2011). Supralinear events, such as dendritic spikes, can be inhibited in an all-or nothing manner by the activation of local inhibitory interneurons (Müller et al., 2012). Additionally, network-state dependent short-term plasticity and external modulation of inhibitory interneurons by the MSDB could change the balance between excitation and inhibition on dendrites dynamically (King et al., 1998; Pouille and Scanziani, 2004; Leão et al., 2012; Müller et al., 2012).

A fundamental task in understanding interneuron diversity is to link structural aspects to physiological functions from the level of the single cell to the network activity in the behaving animal. In the following chapters we attempt to shed light on both, structural and functional aspects with the aim to elucidate the function of two dendrite-innervating interneurons: the O-LM and bistratified cells, in the hippocampal network during behavior.

## STRUCTURAL CHARACTERIZATION OF O-LM AND BISTRATIFIED INTERNEURONS

A characteristic feature of hippocampal interneurons is their morphological diversity (Freund and Buzsáki, 1996). In this section, we will discuss the structural features and molecular markers of O-LM and bistratified cells, highlighting differences and similarities that may contribute to their function.

### ORIENS-LACUNOSUM MOLECULARE CELLS

**FIGURE 1:**
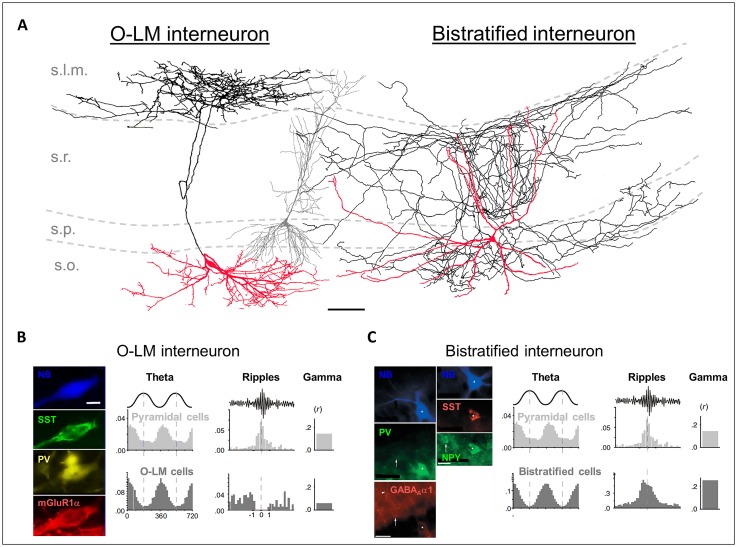
**(A)** Reconstructions of an *in vivo* recorded neurobiotin-labeled (NB) O-LM interneuron (leftmost cell) and a bistratified interneuron (rightmost cell). Red: somata and dendrites; black: axons. The gray cell in the middle is a reconstructed pyramidal neuron for comparison. Scale bar: 100 μm. **(B)** Left panels: the NB O-LM interneuron is immuno-positive for somatostatin (SST), parvalbumin (PV), and mGluR1α. Right panels: *in vivo* firing probability histograms of pyramidal cells and of O-LM interneurons during theta and ripple oscillations and the gamma modulation depth of the firing. **(C)** Left panels: the NB bistratified interneuron is immuno-positive for PV, SST, NPY, and GABA_A_α1. Right panels: *in vivo* firing probability histograms of pyramidal cells and of bistratified interneurons during theta and ripple oscillations and the gamma modulation depth of the firing. [**(A,B)** are modified from Tukker et al. (2007); **(A,C)** from Klausberger et al. (2004), and **(B,C)** from Klausberger and Somogyi (2008) with permissions].

In 1893, Ramón y Cajal described neurons with large somata located in the s.o., near the border to the alveus, with axons ascending to stratum lacunosum moleculare (s.l.m.). This is probably the earliest description of the interneuron type today known as O-LM cells. They have horizontal dendrites, which possess filopodial appendices and span the s.o. but spare the other layers of the hippocampal CA1 region (**Figure 1A**; Freund and Buzsáki, 1996). It has been estimated that in total 1640 O-LM interneurons can be found in CA1, which is 4.3% of all CA1 interneurons (Bezaire and Soltesz, 2013).

**FIGURE 2:**
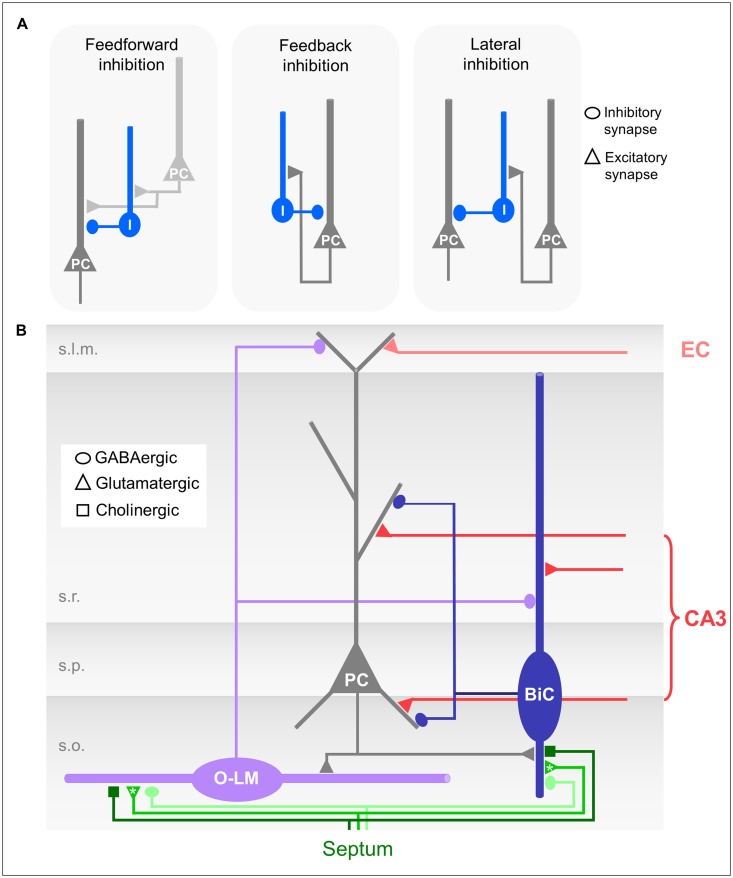
**(A)** Schematic drawings of different forms of microcircuit organizations. Dark gray neuron: CA1 pyramidal cell (PC); light gray: CA3 pyramidal cell; blue: CA1 interneuron. **(B)** Schematic drawing of the CA1 microcircuit involving dendrite-innervating O-LM and bistratified interneurons and septal modulatory innervation. Asterisks indicate likely but unproven connections.

O-LM cells receive excitatory glutamatergic input from the axon collaterals of CA1 pyramidal neurons, and hence fulfill the morphological requirements for feedback or recurrent inhibitory interneurons (**Figures 2A,B**; Sun et al., 2014). Interestingly, the recurrent axon collaterals of the pyramidal cells in the CA3 region are not restricted to s.o. but extend further into the s.r. Accordingly, the dendrites of CA3 O-LM interneurons also extend into this layer following their presynaptic partners in the feedback loop (Gulyás et al., 1993).

Although a comprehensive picture on GABAergic innervation of O-LM cells by other interneurons is not known, there is evidence that these cells are innervated at least by CA1 interneurons specifically innervating other interneurons (ISIs), putative bistratified cells (Leão et al., 2012), and other O-LM interneurons (Acsády et al., 1996; Ferraguti et al., 2004; Kogo et al., 2004).

The axons of the O-LM cells target pyramidal neurons (89%) and interneurons (11%; Katona et al., 1999; Bezaire and Soltesz, 2013). Sik et al. (1995) were the first to recover a full morphology of an *in vivo* filled O-LM cell. They reported a rather compact axonal arbor forming 16 874 boutons, predominantly in the s.l.m., where the pyramidal neuron tuft dendrites are located, and only a small amount in the s.o. (7% of all terminals), where the basal dendrites of CA1 pyramidal neurons are located (**Figure 1A**). It has been estimated that there are in total 77 O-LM synapses per pyramidal cell and that a single O-LM cell makes 10 synapses per connection (Maccaferri et al., 2000; Bezaire and Soltesz, 2013).

Neurochemical markers to identify O-LM interneurons are SST and the glutamate receptor 1α subunit (**Figure 1B**; Naus et al., 1988; Shigemoto et al., 1996; Maccaferri et al., 2000; Klausberger et al., 2003). Furthermore, recent work showed that the nicotinic acetylcholine receptor α2 subunit (nAChRα2) is expressed on O-LM dendrites with a high specificity (Leão et al., 2012). Thus, antibodies targeting nAChRα2 provide an additional tool to identify O-LM cells.

### BISTRATIFIED CELLS

The bistratified cells in the CA1 region were firstly described by Buhl et al. (1994). Similar to the O-LM cells, bistratified cells are immunopositive for SST and PV; however, the expression intensities are complementary. Bistratified cells additionally express the neuropeptide Y (NPY; **Figure 1C**; Somogyi and Klausberger, 2005). SST and NPY might be released by bistratified cells during high frequency firing, such as that observed during sharp wave-ripple oscillations *in vivo* (see Behavioral Relevance of Dendritic Inhibition; Klausberger et al., 2004; Katona et al., 2014). Their release has been shown to down-regulate the glutamatergic transmission of the Schaffer collaterals, and thus may prevent hyper-excitable, epileptiform activity (Moore et al., 1988; Vezzani et al., 1999; Baraban and Tallent, 2004; Klausberger et al., 2004).

The somata of bistratified cells are located in s.o. (24%), s.p. (70%), and s.r. (6%). It has been estimated that a total of 2210 bistratified interneurons can be found in CA1, which is 5.7% of all CA1 interneurons (Bezaire and Soltesz, 2013). The bistratified cells possess aspiny dendrites, which span all layers of the CA1 region but the s.l.m. (Maccaferri et al., 2000).

Bistratified cells receive feedforward excitatory input exclusively from the Schaffer collaterals and commissural projections (Klausberger et al., 2004). They also participate in the feedback circuitry by receiving excitatory input from CA1 pyramidal neurons via axon collaterals in s.o. (**Figures 2A,B**; Ali et al., 1998).

Bistratified cells receive GABAergic input from other local interneurons including O-LM cells (Buhl et al., 1996; Leão et al., 2012). Furthermore, bistratified cells contain a high number of extrasynaptic GABA_A_ receptors, which indicates that they are strongly regulated by tonic GABAergic inhibition (Baude et al., 2007). A sufficient GABA concentration in the extracellular space for activation of these receptors could be mediated by several mechanisms: synaptic spillover of GABA during high frequency release, volume transmitted GABA or by the action of GABA transporters (Farrant and Nusser, 2005; Oláh et al., 2009). Basket cells show a similarly high number of these extrasynaptic receptors. Both bistratified and basket cells receive strong excitatory input from CA3 pyramidal neurons during ripple oscillations and fire phase-modulated action potentials with very high temporal precision *in vivo* (see O-LM and Bistratified Cells in the CA1 Microcircuit and Behavioral Relevance of Dendritic Inhibition; Klausberger et al., 2003, 2004). It has been hypothesized that the strong tonic inhibitory control of bistratified and basket cells is necessary to integrate the substantial excitatory input they receive during fast oscillations into temporally precise output (Baude et al., 2007).

The axons of the bistratified cells predominately terminate in s.o., s.p., and s.r. (**Figure 1A**; Sik et al., 1995). 96% of the axon terminals are located on basal, apical shaft and oblique dendrites, and only 4% on the somata of pyramidal neurons (Halasy et al., 1996). It has been estimated that a bistratified cell forms in total 15,970 synaptic contacts in CA1, with about 6–10 of them on a single pyramidal neuron (Buhl et al., 1994; Sik et al., 1995; Klausberger et al., 2004; Bezaire and Soltesz, 2013). Thus, one bistratified cell innervates approximately 2500 CA1 pyramidal neurons (Sik et al., 1995).

In summary both O-LM and bistratified interneurons preferentially innervate CA1 pyramidal neuron dendrites. However, they terminate on two very different dendritic domains. Each interneuron subtype is thus specialized to counteract synaptic excitatory drive received from one of the two main excitatory input regions.

## FUNCTIONAL ASPECTS OF O-LM AND BISTRATIFIED INTERNEURONS IN THE CA1 MICROCIRCUIT

In the following paragraphs, we connect the structural characteristics of O-LM and bistratified interneurons with their functional relevance.

### ACTIVATION OF O-LM CELLS

**FIGURE 3:**
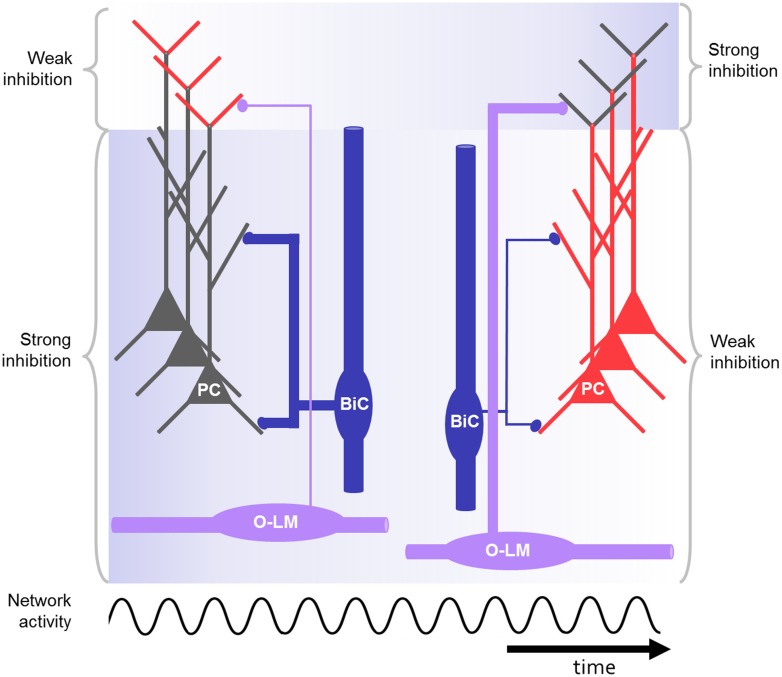
**Schematic drawing of CA1 microcircuit including O-LM and bistratified interneurons during oscillations, e.g., at gamma or theta frequency.** The bistratified interneuron strongly inhibits the proximal dendrites of the pyramidal cell at the beginning (blue background) and is losing its impact during repeated activation of the network. In contrast, the O-LM interneurons display low output at the beginning but gain strength in response to repeated activation. As a result of the dynamic changes of the distal and proximal inhibition in response to rhythmic activation, the excitability in the distal and proximal dendrites is controlled in a network-state dependent manner (red: highly excitable, gray: less excitable). In this way the right cell assembly might become active during rhythmic network activity.

An action potential in a CA1 pyramidal neuron evokes an excitatory post-synaptic potential (EPSP) of about 1 mV in the O-LM cell (mean amplitude: 0.93 ± 1.06 mV; Ali and Thomson, 1998), which alone is likely to be insufficient to cross action potential threshold. However, excitatory input onto the O-LM cells facilitates with repeated firing of the pyramidal neuron. With sufficient inputs O-LM cells generate action potential output with high reliability but low temporal precision (**Figure 3**; Ali and Thomson, 1998; Losonczy et al., 2002; Pouille and Scanziani, 2004; Müller et al., 2012). This facilitation is a prominent feature of the CA1 pyramidal neuron to O-LM interneuron synapses, and is thought to be mediated by the extracellular leucine-rich repeat fibronectin containing protein 1 (Elfn1). This protein is selectively expressed by O-LM cells and regulates the release probability of the pyramidal neuron synapses onto O-LM dendrites (Sylwestrak and Ghosh, 2012). Additionally, the pyramidal neuron to O-LM synapse is likely to be regulated by another target-cell-specific presynaptic mechanism: pyramidal neuron terminals that are presynaptic to mGluR1α expressing interneurons, such as the O-LM cells, contain a ∼10 fold higher amount of the metabotropic glutamate receptor 7 (mGluR7). The high level of mGluR7 results in a frequency dependent auto-regulation of transmitter release in the pyramidal to O-LM cell synapses. Consequently, glutamate release at these synapses is suppressed at high frequencies, and may only be reliably released at lower frequencies, such as in the theta frequency range. However, the expression of the mGluR1α *per se* appears not to be a crucial factor, since this regulatory mechanism could also be observed in transgenic mice lacking these receptors (Shigemoto et al., 1996).

Long positive current injections lead to sustained output of O-LM cells. The action potentials display spike frequency adaptation and are followed by a pronounced afterdepolarizations (Lacaille and Williams, 1990; Zhang and McBain, 1995). Furthermore, long negative current injections into O-LM cells will lead to hyperpolarization followed by depolarization resembling a voltage-“sag.” These characteristics indicate a strong contribution of the hyperpolarization activated cation current (H-current/I_H_; Maccaferri and McBain, 1996; Zemankovics et al., 2010). The expression of the underlying cyclic nucleotide-gated channel can result in spontaneous firing (Maccaferri and McBain, 1996), and equips O-LM cells with an intrinsic resonance frequency at theta (Pike et al., 2000). A preferential tuning of O-LM output to theta frequencies may also be supported by the action of kainate receptors, found in the excitatory synapses terminating on O-LM dendrites in mice (Yang et al., 2006; Goldin et al., 2007).

In summary, a facilitation of glutamate release at the pyramidal neuron to O-LM synapse in response to repeated stimulation, the action of inwardly rectifying AMPA receptors and the intrinsic resonance of O-LM cells at theta frequency, suggest that repeated excitatory input to the O-LM cells is preferentially transformed into sustained output at low frequencies most likely in the theta frequency band. As a consequence, the post-synaptic pyramidal neuron tuft dendrites would receive theta-rhythmic inhibition.

### O-LM CELL MEDIATED INHIBITION

When GABA is released from a presynaptic terminal, receptor channels permeable to chloride and bicarbonate are opened in the post-synaptic dendrite (Kaila, 1994). Under physiological conditions in the adult hippocampus the synaptic reversal potential of these conductances lies below the dendritic membrane potential resulting in a net influx of chloride ions, which hyperpolarizes the dendrite (Kaila, 1994; Glickfeld et al., 2009). The dendritic hyperpolarization propagates and electrotonically attenuates with distance from the input site (Stuart et al., 2008). Therefore, the effectiveness of hyperpolarizing inhibition to counteract coincident synaptic excitation is dependent on the distance between the inhibitory and excitatory input. Additionally, the activation of an inhibitory synapse reduces the local input resistance. While the inhibitory conductances are open, excitatory synaptic currents are short-circuited or shunted. Shunting inhibition is very effective near the site of the inhibitory input and on the path between an excitatory synapse and the action potential initiation site (Koch et al., 1983; Liu, 2004; London and Häusser, 2005; Stuart et al., 2008; Pouille et al., 2013), but may also be potent at off-path locations (Gidon and Segev, 2012). The relative contribution of both inhibitory mechanisms to reduce excitability is dependent on the intracellular chloride concentration, determining the reversal of the GABAergic conductance, and the relative locations of the activated GABAergic and glutamatergic synapses.

When measured at the soma of a CA1 pyramidal neuron, the inhibitory post-synaptic potential (IPSP) elicited by a single O-LM interneuron has a small amplitude and slow kinetics (Maccaferri et al., 2000). Keeping in mind the distance of several hundred micrometers between input site and somatic recording site, it can be assumed that the IPSP is several-fold larger locally at the distal dendrite. Taking into account that in total ∼80 O-LM cell synapses are found per pyramidal neuron (Bezaire and Soltesz, 2013), it is likely that the O-LM mediated inhibition very effectively controls the excitatory input onto the tuft. Interestingly, 20% of the O-LM synapses are formed on dendritic spines (Sik et al., 1995; Katona et al., 1999). It has been demonstrated in neocortical circuits that SST expressing interneurons, forming inhibitory synapses on spines, can control the excitation of individual spines (Chiu et al., 2013). The impact of such inhibitory conductances on a single spine is likely to be spatially restricted, due to the spine neck resistance. In this way, O-LM interneuron-mediated inhibition in CA1 could counteract the excitatory input in a synapse specific manner.

The main termination zone of O-LM cells are the pyramidal neuron dendritic tuft, where excitatory glutamatergic input from the entorhinal cortex (EC) is received. This excitatory input alone is sufficient to evoke place related firing in pyramidal cells (Brun et al., 2002), and is likely controlled by O-LM mediated inhibition. Compared to the oblique dendrites, glutamatergic input to the tuft dendrites evokes EPSPs with a larger NMDA/AMPA ratio (Otmakhova et al., 2002). Additionally, the contribution of stronger, perforated synapses is higher on the tuft dendrites (Nicholson et al., 2006). Together, these synaptic properties counteract the distance dependent attenuation of distal EPSPs. The strong impact of excitatory inputs locally on the distal tuft dendrites could be balanced by the two to three fold higher density of GABAergic synapses observed on tuft dendrites compared to oblique dendrites (Megías et al., 2001). The dendritic filtering of distal post-synaptic potentials is counteracted by the H-current, which is highly expressed in distal dendrites (Magee, 1998). This current curtails the post-synaptic potentials (PSPs) and reduces the input site dependent slowing of the time-course of distal PSPs (Williams and Stuart, 2000). Briefer and more precise PSPs would improve the accuracy of coincidence detection with signals from further proximal sites (Magee, 1998).

In response to clustered excitatory inputs, it has been shown that apical tuft dendrites generate supralinear dendritic events (dendritic spikes), which can overcome the strong distance dependent attenuation (Jarsky et al., 2005). Experimentally, this is usually demonstrated under conditions during which inhibition is blocked (Golding et al., 2001; Jarsky et al., 2005; Takahashi and Magee, 2009). However, inhibition could play a crucial role in controlling the supralinear integration in tuft dendrites. Since inhibition on more proximal dendrites is able to prevent supralinear integration, O-LM mediated inhibition may have a substantial impact on dendritic supralinear integration in the tuft (Lovett-Barron et al., 2012; Müller et al., 2012; Stuart, 2012). We have previously shown that activation of recurrent inhibition, to which O-LM cell are a main contributor, strongly reduces local excitation in the tuft dendrites (Müller et al., 2012).

### ACTIVATION OF BISTRATIFIED CELLS

In contrast to O-LM cells, glutamatergic input onto bistratified cells is not exclusively mediated by CA1 pyramidal neurons via axon collaterals, but also by CA3 pyramidal neurons via the Schaffer collaterals. A single action potential of a CA1 pyramidal neuron was found to evoke an EPSP of 3.4 ± 3.1 mV, when measured at the soma of a bistratified cell (Ali et al., 1998). This input is much larger compared to the excitation an O-LM cell receives from a single CA1 pyramidal neuron. However, O-LM cell dendrites can amplify dendritic input by the recruitment of dendritic sodium channels (Martina et al., 2000) and thereby produce reliable output. Both bistratified and O-LM cells receive phasic glutamatergic synaptic input primarily mediated by calcium permeable AMPA receptors (Oren et al., 2009; Wondolowski and Frerking, 2009; Nissen et al., 2010). In contrast to AMPA receptors on pyramidal neurons or CCK expressing dendrite-targeting interneurons, these calcium-permeable AMPA receptors display inward rectification, i.e., the synaptic currents are suppressed at depolarized membrane potentials. The activation of these calcium permeable AMPA receptors leads to precise calcium influx into the interneuronal dendrites (Topolnik et al., 2005), which can induce calcium-dependent, NMDA receptor independent plasticity at subthreshold membrane potentials (Nissen et al., 2010; Szabo et al., 2012; Camiré and Topolnik, 2014). Thus, these specific glutamate receptors enable bistratified and O-LM cells to respond to specific input patterns with synaptic plasticity, whereas synapses onto other dendrite-targeting interneurons (e.g., CCK expressing interneurons) would remain unaffected by the same input.

In response to repeated firing of presynaptic CA1 pyramidal neurons the excitation in the bistratified interneuron undergoes synaptic depression (**Figure 3**; Ali et al., 1998; Pouille and Scanziani, 2004; Müller et al., 2012). In response to repeated firing of presynaptic CA3 pyramidal neurons, in contrast, the excitatory input onto bistratified cells might facilitate (Wierenga and Wadman, 2003). Also, more complex patterns of short-term plasticity were found in this interneuron type (Losonczy et al., 2002).

When excitation in bistratified interneurons crosses the action potential threshold, non-accommodating action potentials, burst firing and maximum firing rates above 100 Hz can be observed. This allows them to perform high frequency discharges, e.g., during ripple oscillations observed *in vivo* (Buhl et al., 1996; Pawelzik et al., 2003; Klausberger et al., 2004; Baude et al., 2007; Katona et al., 2014). As in O-LM cells, the firing mode of bistratified interneurons may be influenced by the action of H-currents, which imposes a subthreshold resonance at 2–6 Hz onto these cells (Zemankovics et al., 2010).

### BISTRATIFIED CELL MEDIATED INHIBITION

The IPSCs evoked by bistratified interneurons in pyramidal neuron oblique and basal dendrites are larger compared to O-LM mediated IPSCs, when measured at the soma (∼50 pA compared to 2.6 pA; Maccaferri et al., 2000). The large amplitude of a somatically measured bistratified cell evoked IPSP (-0.86 ± 0.55 mV; Pawelzik et al., 1999) could partially be explained by the proximal input site, which results in less attenuation along the dendrite to the somatic recording site. It has been estimated that a single pyramidal neuron receives inhibitory input from ∼10 different bistratified interneurons making on average ∼10 synapses per connection, preferentially on the small caliber dendrites in s.o. and s.r. of the pyramidal neurons (Klausberger et al., 2004; Bezaire and Soltesz, 2013). Therefore, it can be assumed that bistratified interneurons provide strong dendritic inhibition and can thereby control the transformation of CA3 excitatory input to action potential output in CA1 pyramidal neurons (Klausberger et al., 2004). Inhibitory synapses in general and those formed by a single bistratified cell are unlikely to be clustered on a specific pyramidal neuron dendritic branch. Consequently, the idea that single interneurons may functionally veto excitation on a single dendritic branch is not supported; rather a global and distributed inhibitory innervation of dendrites can be expected (Buhl et al., 1994; Liu, 2004). However, some evidence for a selective suppression of specific dendritic compartments by the feedforward interneuron population, including bistratified cells, has recently been provided by single cell voltage imaging experiments (Willadt et al., 2013).

The basal and proximal oblique dendrites of the CA1 pyramidal neurons are capable of integrating excitatory input supralinearly in form of dendritic spikes (Stuart et al., 1997; Golding and Spruston, 1998; Gasparini et al., 2004; Losonczy and Magee, 2006). Dendritic spikes have been shown to be effective triggers of action potential output and synaptic plasticity (Golding and Spruston, 1998; Golding et al., 2002; Ariav et al., 2003; Remy and Spruston, 2007; Müller et al., 2012). One important requirement for the induction of dendritic spikes is the temporal and spatial clustering of excitatory input on dendritic branches (Gasparini et al., 2004; Losonczy and Magee, 2006). These conditions are likely to be found during sharp wave-ripples *in vivo* (Csicsvari et al., 2000). It has been shown in experiments using simultaneous GABA and glutamate iontophoresis or uncaging that the activation of GABAergic synapses, on the path between the dendritic spike initiation and the somatic recording site, suppresses dendritic spiking (Lovett-Barron et al., 2012; Müller et al., 2012). Bistratified interneurons are ideally suited to block dendritic spikes by providing strong inhibition onto small caliber dendrites.

In experiments using optical activation and cell type-specific pharmacogenetic silencing, dendritic inhibition in general was more effective in shunting dendritic spikes and action potential burst firing than somatic inhibition, and determined the gain of the input–output transformation in these neurons (Lovett-Barron et al., 2012). In these experiments, the surprisingly small effect of somatic inhibition on the input–output transformation in pyramidal neurons was explained by interneuron–interneuron connections. Consequently, the silencing of soma-targeting interneurons resulted in disinhibition of dendrite-innervating interneurons so that these still reduced pyramidal neuron output (Lovett-Barron et al., 2012).

When a subpopulation of recurrent interneurons including bistratified cells is activated by alvear stimulation, inhibition onto oblique and basal pyramidal neuron dendrites is evoked. This is sufficient to block dendritic spiking and to narrow the time window for excitatory signal integration and action potential generation. However, when activated repeatedly at theta frequency, the recurrent inhibition on proximal dendrites is reduced, due to the depressing excitatory input from the CA1 pyramidal neurons to the interneurons innervating these dendrites, including bistratified cells (Pouille and Scanziani, 2004; Müller et al., 2012). Subsequently, the inhibition of dendritic spiking mediated by these interneurons is less effective. A subset of dendritic branches however, which give rise to strong dendritic spikes, can escape inhibitory control (Müller et al., 2012). Therefore, the activation of a presynaptic CA3 pyramidal neuron subpopulation is likely to generate action potential output in CA1 pyramidal neurons, either when it provides clustered input to a specific dendrite, which generates a strong dendritic spike; or when the impact of the inhibitory microcircuit is decreased as a result of synaptic depression during repetitive, rhythmic activity.

### O-LM AND BISTRATIFIED CELLS IN THE CA1 MICROCIRCUIT

In the CA1 region both feedback and feedforward inhibitory circuits are found (see Structural Characterization of O-LM and Bistratified Interneurons, Activation of O-LM Cells, and Activation of Bistratified Cells). The two forms of microcircuit organizations have functional consequences for dendritic inhibition. In feedforward microcircuits inhibitory interneurons are activated by afferent input, e.g., CA3 pyramidal neurons (**Figure 2A**) and subsequently inhibitory and excitatory inputs coincide in the post-synaptic neuron, e.g., the CA1 pyramidal neurons (Karnup and Stelzer, 1999; Pouille and Scanziani, 2001). Thereby, a high number of excitatory inputs could be balanced by stronger simultaneously activated feedforward inhibition. Feedforward inhibition thus can serve to increase the dynamic range of excitatory input integration (Pouille et al., 2009, 2013). The postsynaptic neuron remains sensitive to small excitatory inputs, and at the same time saturation is prevented so that strong excitatory inputs can still be discriminated. Feedforward inhibition might prevent the post-synaptic population from firing at all; feedback inhibition however, requires output from the CA1 pyramidal neurons. Consequently, O-LM interneurons, which are exclusive feedback interneurons, are not able to inhibit CA1 pyramidal neuron output *per se*, but are able to control the duration of the CA1 activation and to modulate the relative contribution of excitatory EC input to output generation.

Bistratified cells, in contrast, take part in both, feedforward and feedback microcircuits (**Figure 2A**). Due to the different input dynamics in response to sustained activation by either CA3 or CA1 pyramidal neurons (see Activation of Bistratified Cells; Wierenga and Wadman, 2003), the extent to which bistratified cells engage in either feedforward or feedback inhibition might depend on the duration and frequency of the excitatory inputs they receive from the presynaptic population.

In its classical meaning, feedback inhibition in CA1 requires action potential output of a CA1 pyramidal neuron population. When firing a population of CA1 pyramidal neurons and thus activating a subset of feedback interneurons by using alvear stimulation, simultaneously recorded pyramidal neurons receive IPSPs. Firing of the recorded CA1 pyramidal neuron itself in response to stimulation is scarce and has not been reported (Pouille and Scanziani, 2004; Müller et al., 2012). The lack of action potentials in the cell, which actually receives inhibition, implies that in the case of predominant lateral inhibition persistent excitatory output by a CA1 pyramidal neuron subpopulation could lead to inhibition of a different subpopulation of CA1 pyramidal neurons (Jonas and Buzsáki, 2007; Dupret et al., 2013).

Likewise, it is not clear how strictly the concept of feedforward inhibition is implemented in the connections between CA3 and CA1, particularly, whether a CA3 cell population inhibits exclusively the CA1 cell population that is simultaneously excited. Or if feedforward inhibition is a tool to increase the contrast between different cell groups, regarding their excitability. The different cell groups separated by inhibition could serve as neuronal assemblies, which encode distinct pieces of information (Geisler et al., 2007; Buzsáki, 2010). Neuronal assemblies might be formed, e.g., during spatial navigation of the animal, where participating CA1 principal neurons show correlated phase precession in subsequent theta cycles (O’Keefe and Recce, 1993; Skaggs and McNaughton, 1996; Maurer et al., 2006a; Geisler et al., 2007). Also interneurons participate in place- and phase-related firing and may support assembly formation (McNaughton et al., 1983; Kubie et al., 1990; Marshall et al., 2002; Maurer et al., 2006b; Geisler et al., 2007). Soma-innervating fast spiking interneurons, such as basket cells, have been identified as ideal candidates to serve this function (Geisler et al., 2007). However, bistratified cells have many similar properties to basket cells, they both: are recruited in the feedforward and feedback microcircuitry (Ali et al., 1998; Wierenga and Wadman, 2003), can fire high frequencies (Buhl et al., 1996), respond similarly to gamma rhythmic repeated input (Pouille and Scanziani, 2004), their firing *in vivo* is strongly modulated by gamma oscillations (see Behavioral Relevance of Dendritic Inhibition; Klausberger and Somogyi, 2008), and they both receive strong excitatory input during sharp-waves (see Bistratified Cells; Klausberger et al., 2003, 2004). Therefore, bistratified cells could be the dendrite-targeting complement to basket cells serving a similar function in the network with respect to assembly formation. Since O-LM interneurons show no phase precession it is less likely that they support the formation of assemblies encoding correlated spatial information (Geisler et al., 2007).

The separation of cell assemblies may not only be a result of the fixed wiring between pyramidal neurons and local interneurons. As described in Section “Activation of O-LM Cells and Activation of Bistratified Cells,” the synapses between pyramidal cells and O-LM or bistratified interneurons exhibit short-term plasticity in response to certain input patterns, e.g., theta or gamma rhythmic input from presynaptic pyramidal neurons (**Figure 3**; Ali and Thomson, 1998; Ali et al., 1998; Pouille and Scanziani, 2004; Müller et al., 2012; Sylwestrak and Ghosh, 2012). This could lead to dynamic changes of the synaptic weights during different activity states of the network. The changed impact of distinct interneuron groups, acting on specific dendritic domains, could help to separate cell assemblies by tweaking their activity (Buzsáki, 2010).

In conclusion, the effect of inhibition on dendritic excitation is mainly determined by the domain specificity of the axonal arborization of the interneuron subtype, the dynamic and the resulting synaptic strength of dendritic inhibition, and by the pattern of the excitatory input. Among the diverse interneuron subpopulations the synapses formed by dendrite-targeting interneurons are placed in strategic positions to counteract dendritic excitation, to prevent dendritic supralinearities and, as a consequence, action potential output (Buzsáki et al., 1996; Miles et al., 1996; Takahashi and Magee, 2009; Lovett-Barron et al., 2012; Müller et al., 2012). O-LM and bistratified cells provide pathway specific inhibition, and thus may play an important role in matching the direct sensory input from the EC to the information that is relayed through CA3. A temporally coordinated interaction of the two excitatory pathways may be essential for CA1 pyramidal neurons to subserve the comparator function (Vinogradova, 2001; Takahashi and Magee, 2009). The pathway specific inhibition by O-LM and bistratified cells changes dynamically when the network is repeatedly activated (Pouille and Scanziani, 2004; Müller et al., 2012). A dynamic change from predominant proximal dendritic to predominant distal dendritic inhibition could lead to preferential processing of input from one pathway over the other, depending on the network-state. Additionally, O-LM cells directly inhibit feedforward interneurons, including bistratified cells. Thus, activating O-LM cells not only inhibits the perforant path excitatory input, but also amplifies the impact of the Schaffer collateral excitatory input. This latter effect is due to the reduction in feedforward inhibition by bistratified cells and other interneurons (Leão et al., 2012). This mechanism could consolidate the activity dependent weighting of the two excitatory inputs to the CA1 pyramidal neuron dendritic domains. Furthermore, the perforant path excitation has been shown to increase Schaffer collateral plasticity depending on the relative input timing (Remondes and Schuman, 2002). This gating mechanism could be controlled by the pathway specific dynamic inhibition provided by O-LM and bistratified cells. The recruitment in different microcircuits in CA1 could allocate bistratified and O-LM cells with distinct roles during specific network activities *in vivo* (see Behavioral Relevance of Dendritic Inhibition).

## SEPTAL MODULATION OF O-LM AND BISTRATIFIED CELL MEDIATED INHIBITION

During theta oscillations, the activity of hippocampal interneurons is modulated by the MSDB (Freund and Antal, 1988; Tóth et al., 1997; Sun et al., 2014). Different septal cell types discharge at theta frequencies *in vivo* and project to the hippocampal formation via the fimbria/fornix fiber bundle (King et al., 1998). One main projection is cholinergic and terminates specifically on hippocampal interneurons (Bland and Bland, 1986; Frotscher et al., 1986). The action of acetylcholine is mediated by muscarinic and nicotinergic receptors. When the muscarinic receptor agonist carbachol is bath applied to a hippocampal slice in high concentrations, cells depolarize and action potential output at theta frequency is facilitated (Bland et al., 1988). CA1 interneurons are also activated by application of muscarinic receptor agonists and consequently more IPSPs in pyramidal neurons can be observed (Pitler and Alger, 1992). In O-LM interneurons the pharmacological activation of muscarinic receptors leads to enhanced action potential firing (Lawrence et al., 2006b). Furthermore, the output reliability and precision of action potential firing in response to theta patterned input is improved (Lawrence et al., 2006a). Recent work by Nagode et al. (2011) confirmed these results using an optogenetic approach.

Since muscarinic receptors are also found in presynaptic terminals they may additionally modulate transmitter release. Zheng et al. (2011) demonstrated that glutamatergic EPSPs in putative O-LM interneurons were reduced in amplitude when muscarinic receptors were activated. In contrast, interneurons located in s.r., including putative bistratified cells, received increased glutamatergic input, emphasizing the diversity of cholinergic effects on hippocampal interneurons.

Upon electrical stimulation of the fimbria/fornix fiber bundle, in a septo-hippocampal slice preparation, O-LM and bistratified cells show clearly discrete responses: the O-LM cells depolarize, whereas the bistratified cells are either unaffected or show a biphasic response, consisting of first hyper- and subsequent depolarization. However, it has to be noted that by stimulating these afferents electrically, also other neurotransmitters may be released, potentially underlying some of the diverse response patterns (Widmer et al., 2006). Leão et al. (2012) showed that, when evoking nicotinergic transmission by fimbria/fornix stimulation, O-LM cells received fast excitatory currents mediated by the nicotinic receptors containing the α2 and α7 subunit. These cholinergic projections from the MSDB can excite O-LM cells sufficiently to cross action potential threshold (Lovett-Barron et al., 2014). There is evidence that also bistratified interneurons might be activated by acetylcholine receptors containing the α7 subunit (Buhler and Dunwiddie, 2002; Son and Winzer-Serhan, 2008).

The effect of muscarinic receptor activation is slow (Hasselmo and Fehlau, 2001; Buzsáki, 2002), and thus it is likely to increase the overall excitability of pyramidal neurons and interneurons in CA1 during theta episodes (Madison et al., 1987). In contrast, nicotinergic input from the MSDB could rhythmically recruit CA1 interneurons and therefore set the pace for synchronous CA1 interneuron activity (Freund and Antal, 1988; Buzsáki, 2002; Cobb and Davies, 2005; Hangya et al., 2009). To fully understand the role of the septo-hippocampal pathway on hippocampal theta oscillations, it is necessary to know, which specific interneuron subtypes are targeted, and how muscarinic and nicotinergic receptors act on these cells during behavior.

Interestingly, in a recent study using rabies virus mediated tracing, excitatory glutamatergic input onto CA1 inhibitory interneurons from the MSDB was found (Sun et al., 2014). The functional implications of this additional extrinsic glutamatergic input in general and specifically for bistratified and O-LM interneurons are not yet understood. Furthermore, O-LM and bistratified cells are targeted by axons from GABAergic projection neurons located in the MSDB (Gulyás et al., 1990; Chamberland et al., 2010). These GABAergic projections provide rhythmic inhibition onto O-LM and bistratified neurons during theta oscillation and strengthen their synchronous theta rhythmic firing observed *in vivo* (Tóth et al., 1997; Bland et al., 1999; Klausberger, 2009). A potential role of septal GABAergic projection neurons innervating CA1 interneurons was pointed out by Kaifosh et al. (2013), who imaged the calcium signals in boutons from septal GABAergic projection neurons onto CA1 interneurons located in s.o., including putative O-LM cells. In response to diverse sensory stimuli with different intensities the septal cells generated GABAergic theta modulated output onto the CA1 interneurons and provided information about the stimulus intensity but not its identity.

Clearly, there are still open questions about the specific modulation of O-LM and bistratified interneurons by the MSDB during behavior. Dendrite-innervating interneurons are powerful targets for MSDB modulation because of their potential to switch weights of the two major excitatory input pathways terminating on CA1 pyramidal neurons. The availability of a diverse modulatory toolset, extending the repertoire by which integration on different dendritic domains can be fine-tuned by the MSDB, suggests that modulation of dendritic inhibition may serve specific functions during different behavioral states.

## BEHAVIORAL RELEVANCE OF DENDRITIC INHIBITION

Theta oscillations are observed in the hippocampus during movement, REM sleep and states of arousal (Grastyán et al., 1965; Jouvet, 1969; Vanderwolf, 1969; O’Keefe and Nadel, 1978). In the CA1 field of the hippocampus, theta oscillations are thought to be regulated by cholinergic and GABAergic projections from the MSDB (see Septal Modulation of O-LM and Bistratified Cell Mediated Inhibition; Freund and Antal, 1988; Stewart and Fox, 1990; Buzsáki, 2002). In early *in vivo* recordings, it became clear that not only the principal cells in the hippocampus participate in rhythmic theta firing during behavior, but also that putative interneurons fired with high fidelity in relation to the theta rhythm (Ranck, 1973).

O-LM and bistratified cells display theta rhythmic firing under urethane anesthesia *in vivo*, and both discharge preferentially at the trough of the theta oscillations (**Figures 1B,C**; Klausberger, 2009). This phase relationship has also been observed in awake rodents (Varga et al., 2012; Katona et al., 2014). How could a similar phase coupling of O-LM and bistratified cells during theta oscillations influence signal integration on CA1 pyramidal neuron dendrites? Synchronous discharge of O-LM and bistratified cells would hyperpolarize the CA1 pyramidal neuron dendrites in both major input domains. Thereby the membrane potential and the integration of excitatory inputs on the whole dendritic tree would become phase-modulated (Kamondi et al., 1998; Klausberger et al., 2004). This could create windows of opportunity for excitatory synaptic inputs to initiate action potential output (Csicsvari et al., 1999). Furthermore, it has been hypothesized that the rhythmic hyperpolarization during theta oscillations, mediated by dendrite-innervating O-LM and bistratified cells, could facilitate the recovery of dendritic low-threshold calcium channels from inactivation (Magee and Johnston, 1995; Klausberger et al., 2004). In this way, synchronized dendritic hyperpolarization could globally decrease the excitability in the pyramidal neuron population, but maximize the bursting probability in a few cells that receive strong excitatory input (Klausberger et al., 2004). This hypothesis is supported by the finding that a place-cell is likely to fire bursts at the opposite theta phase compared to when O-LM and bistratified cells fire preferentially (O’Keefe and Recce, 1993; Harris et al., 2001; Buzsáki, 2002; Klausberger et al., 2004). Taken together, theta rhythmic dendritic inhibition may not only regulate dendritic excitation by inhibiting synaptic input, but also dynamically facilitate action potential output by the regulation of intrinsic ionic conductances.

Interestingly, the recruitment of O-LM and bistratified cells into other types of oscillatory activity is fundamentally different. O-LM cells fire few bursts *in vivo*, decrease their firing when the animal is sleeping, and show no phase-coupled firing to gamma oscillations. Bistratified cells, in contrast, fire at high rates during sleep, increase their firing during sharp wave-ripple episodes and are likely to exhibit burst firing. (**Figures 1B,C**; Klausberger et al., 2003, 2004; Tukker et al., 2007; Klausberger and Somogyi, 2008; Katona et al., 2014). Furthermore, it was demonstrated that bistratified cells show a specifically strong gamma modulated firing (Tukker et al., 2007). Gamma oscillations (25–140 Hz) in the hippocampus are generated by inputs from CA3 and the EC and are often nested in slower theta oscillations. They are thought to coordinate the activation of neuronal assemblies on short time scales, e.g., during memory retrieval. IPSPs onto pyramidal neurons have been identified to be the main source of the gamma oscillation in the local field potential. These IPSPs are supposed to be generated by basket and bistratified cells (Soltesz and Deschênes, 1993; Penttonen et al., 1998; Colgin, 2011). Bistratified cells receive strong excitatory input from CA3, and are thus likely to transmit the CA3 dependent component of gamma to the CA1 region.

As hypothesized above, inhibition could provide a tool to select specific cell assemblies (see O-LM and Bistratified Cells in the CA1 Microcircuit; **Figure 2A**). The interneurons activated by one CA1 pyramidal neuron assembly could inhibit another, distinct CA1 assembly. A recent study suggests that the activation of interneurons in CA1 contribute to the reorganization of pyramidal neuron assemblies, which plays an important role during spatial learning tasks. Dupret et al. (2013) demonstrated that the establishment of a behaviorally relevant neuronal assembly involves the modification of inhibitory microcircuits. The excitatory connections from CA1 pyramidal neurons onto local interneurons change dynamically according to the activity of the presynaptic assemblies in CA1 (Pouille and Scanziani, 2004). Since they are connected in this feedback/lateral inhibitory loop, the interneurons may dynamically adjust the recruitment of specific pyramidal neuron assemblies via short-term plasticity of the pyramidal cell to interneuron synapses. The specific interneuron subtypes involved in the assembly formation is not yet clear; however, the location of the recorded interneurons in the s.p. suggests that O-LM cells are unlikely to contribute, at least in the tested behavioral paradigm (Dupret et al., 2013).

The specific roles of dendrite- versus soma-innervating interneurons were investigated by Royer et al. (2012). They performed *in vivo* recordings of the local field potential and unit responses during locomotion on a treadmill in head restrained mice. Using optogenetic tools, they specifically reduced either the inhibition mediated by PV or SST positive interneurons in CA1. Reducing the availability of SST positive putative dendrite-innervating interneurons had the strongest effect on the spatially modulated firing of pyramidal neurons. Dendritic inhibition reduced both the overall firing and the burst firing, but not the phase-relation of pyramidal neuron firing. A similar effect of dendritic inhibition had also been demonstrated in earlier *in vitro* experiments (Miles et al., 1996). Putative soma-innervating PV-positive interneurons were more likely to be recruited at the rising phase of the theta cycle, dendrite-innervating interneurons at the descending phase. The authors speculated that this could be due to the delayed recruitment of interneurons innervating the distal tuft dendrites of pyramidal neurons. As described above this delay may be caused by the weak but short-term facilitating synaptic transmission between pyramidal neurons and O-LM cells (**Figure 3**, see Functional Aspects of O-LM and Bistratified Interneurons in the CA1 Microcircuit; Pouille and Scanziani, 2004; Müller et al., 2012; Royer et al., 2012).

A recent pioneering *in vivo* study demonstrated how the pathway specific inhibition mediated by putative O-LM cells may be relevant for behavior. In a fear conditioning experiment, Lovett-Barron et al. (2014) associated an environmental context with an aversive event. One important finding was that the discrete sensory information about the environment is conveyed to the CA1 pyramidal neurons’ dendritic tuft from the EC. This sensory information is integrated with context related information, contributed by the CA3 input to the proximal dendrites of CA1 pyramidal neurons. The authors find that the association with an aversive stimulus can only be successful when the discrete sensory information to the tuft dendrites is excluded by inhibition. This function could be achieved by the O-LM cells, which selectively inhibit the information from the EC about the discrete sensory cues (Lovett-Barron et al., 2014). To examine this idea, Lovett-Barron et al. (2014) optogenetically targeted SST positive interneurons. Indeed, inactivation of SST positive interneurons during conditioning and recall prevented learning of the association. Since only a subpopulation of the SST positive neurons were active during the aversive stimulus, they concluded that this specific task is managed by a subpopulation of SST positive interneurons, most likely the O-LM cells. Additionally, they demonstrated that SST positive, putative O-LM cells, could be driven by cholinergic excitation from the MSDB. The cholinergic drive was essential to manage the learning task, and could explain the rather puzzling finding that the O-LM cells were activated, despite the CA1 pyramidal neurons, providing the only glutamatergic innervation of O-LM cells, did not fire during that specific task (Lovett-Barron et al., 2014). Thus, the additional excitatory cholinergic drive from the MSDB onto O-LM interneurons in CA1 appears to be necessary for the association during a fundamental learning task such as fear conditioning.

In the same study, the authors suggested that other dendrite-innervating SST positive interneurons, such as the bistratified cells serve a different function during learning (Lovett-Barron et al., 2014). Bistratified interneurons are well suited to specifically counteract Schaffer collateral excitatory input due to their unique preference for small to medium size dendrites (Klausberger et al., 2004). During sharp wave-ripple episodes *in vivo*, the CA3 excitatory input is synchronous and strong, which may evoke dendritic spiking and associated synaptic plasticity (Remy and Spruston, 2007; Remy et al., 2009). Specifically, bistratified cells fire high frequencies and appear to be directly activated by CA3 input, since they increase their firing already before the ripples (Klausberger et al., 2003). In this way bistratified cells may counteract the synchronous excitatory input during sharp-wave ripples, which are thought to be important for memory consolidation (O’Neill et al., 2010).

## CONCLUSION

Even when focusing on only two of the at least 21 different subtypes of GABAergic interneurons in the CA1 region of the hippocampus (Klausberger and Somogyi, 2008), the high degree of functional specialization of interneurons becomes obvious. A general motif of O-LM and bistratified cell function in the CA1 microcircuit is the pathway specificity of the dendritic inhibition they provide. It enables them to balance the impact of excitation from the EC and CA3 on pyramidal neuron dendrites selectively. The comparison of inputs from these two major pathways is a characteristic operation of CA1 pyramidal neurons and may be crucially supported by the activity of O-LM and bistratified cells. Furthermore, the microcircuits in the hippocampus could serve to form distinct neuronal assemblies encoding for similar information. Compared to O-LM cells, bistratified interneurons are the more likely candidate to participate in this function of the hippocampal microcircuits.

Both O-LM and bistratified interneurons are controlled by the septo-hippocampal pathway. Cholinergic control by the MSDB is relevant to master certain behavioral tasks. However, understanding the specific roles of the different cell types in the MSDB projecting onto CA1 interneurons is a very important step, which still requires extensive research.

In the behaving animal during place-related theta oscillations, O-LM and bistratified cells may work together, as they fire at the same phase, and thereby generate global dendritic inhibition. During gamma oscillations, in contrast, only bistratified cells are likely to play an important role in transmitting the CA3 dependent gamma component to CA1. O-LM cells on the other hand, seem to undertake a unique function during fear learning. Here, their innervation pattern enables them to selectively silence extrinsic input from the EC.

Although impressive progress has been made in elucidating the function of specific interneuron subtypes during behavior, most of them are still not functionally characterized. New experimental tools, such as optogenetic targeting of interneurons expressing unique marker-sets, open up the possibility to control selective interneurons during behavioral tasks. Optogenetic targeting allows researchers to investigate the modulation of interneurons by remote inputs, e.g., inputs from the MSDB, during behavior. Additionally, genetically expressed calcium indicators in specific interneuron subtypes will allow the recording of their activity patterns *in vivo*, and aid in the understanding of how interneuron activity contributes to specific behaviors.

## Conflict of Interest Statement

The authors declare that the research was conducted in the absence of any commercial or financial relationships that could be construed as a potential conflict of interest.
